# Supplementary material for: Attenuating the DNA damage response to double-strand breaks restores function in models of CNS neurodegeneration
Source: Brain Commun. 2019 Jul 2;1(1):fcz005. doi: 10.1093/braincomms/fcz005 (PMC7425387; doi:10.1093/braincomms/fcz005)
Supplement: fcz005_Supplementary_Data [file fcz005_supplementary_data.zip › Supplementary Materials to UPLOAD.docx]

**Supplementary Fig. 1.** Image processing of DNA double strand breaks in *Drosophila* neurons. 41 x 41 x 13.5 μm volumes of adult central brain neurons stained with anti-pH2Av were visualized by confocal microscopy with 0.45 m step sizes in z. A 63x N.A. 1.2 water immersion lens was used mounted on an inverted Zeiss LSM880 microscope and the Airy scan module used with super-resolution settings to improve resolution. Anti-pH2Av shows a uniform nuclear fluorescence both in our hands and in a previous report (Bellesi *et al.*, 2016). Left panels show the projected z-series of images without adjustments to contrast or brightness. The right panels are the identical images processed to remove the low intensity fluorescence by resetting the black level to display only the bright pH2Av^+^ foci. Control neurons have a single pH2Av^+^ focus corresponding to the nucleolus organizing region. Arrowheads point to clusters of foci in neurons expressing Aβ_1-42_. Scale bars = 10µm.

**Supplementary Fig. 2.** Genetic depletion of the MRN complex is neuroprotective in *Drosophila* neurodegeneration models. (**A**) Quantification of Tau.R406W expression in the heads of flies by Western blot. Flies were developed at 18 °C to inhibit expression of Tau then decapitated 0, 7, 14 or 21 d after shifting to 29 °C. Tau proteins levels are not altered in *nbs^-/+^* heterozygotes. Phosphorylation of Tau at Thr181 is also unchanged. Quantification of Tau levels normalized to actin and the Tau:pTau ratio is shown below (n = 3; mean ± SEM; ANOVA with Dunn’s post-hoc test). (**B**) Negative geotaxis climbing assay. Flies expressing Htt.Q128 show premature decline in climbing ability. This is partially suppressed in flies heterozygous for a null *rad50^EP1^* allele (p=0.0372) despite *rad50* heterozygous flies already showing reduced climbing ability *vs*. the *w^1118^* control (p=0.0007). Lines were fitted using non-linear regression and compared by extra sum-of-squares F-test. (**C**) Expression of Htt.Q128 in the eye leads to loss of pigment-containing cells in the retina after 7 weeks. Pigment loss is largely prevented in flies *rad50^-/+^* flies. (**D**) and (**E**) Negative geotaxis climbing assays of flies expressing two forms of tandem Aβ_1-42_ expression with no flexible linker or with a 12-amino acid flexible linker (**D**) in adult neurons (Speretta *et al.*, 2012). Flies climbed in vials with three marked zones. Aβ_1-42_ expressing (red) flies were compared to Aβ_1-42_; *nbs^1/+^* flies (blue) on the days indicated after shifting to 29 °C to induce expression. Comparison was by χ^2^ test (* indicates P<0.05). (**F**) Circadian periodicity analyzed by cosinor analysis shows similar results to the CLEAN spectral analysis shown in Fig. 1F (Rosato and Kyriacou, 2006). Comparison by ANOVA with Tukey’s post-hoc test; *P* values and *n* are indicated.


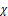


**Methods for Tau quantification in (A).** 15 adult flies per genotype and time point were decapitated with a razor blade then homogenized in 150 μl RIPA buffer (New England Biolabs) supplemented with protease and phosphatase inhibitor cocktails (both Merck). Homogenization used a MP Biomedicals FastPrep blender running at 6.0 m/s for 20 s in screwcap tubes containing matrix C garnet beads (MP Biomedicals). Lysates were clarified by centrifugation at 12000 x g for 5 min at 4 °C then 6X Laemlli buffer + 50 mM DTT was added and the samples boiled for 5 min. Proteins were separated on 12% Tris.Glycine SDS-PAGE gels and transferred to PVDF membranes. Primary antibodies used were: rabbit anti-total Tau (1:1.000; DAKO); mouse anti-Tau pThr181 (1:500; ThermoFisher) and mouse IgM anti-actin JLA20 (1:5.000; Developmental Studies Hybridoma Bank). Secondary antibodies used were: HRP-conjugated horse anti-mouse IgG and HRP-conjugated horse anti-rabbit IgG (both 1:10,000; New England Biolabs; note: the anti-mouse IgG secondary antibody cross-reacts with mouse IgM). Membranes were visualized using a Vilber Fusion FX scanner. Total Tau levels were normalized to actin for each sample and phosphorylated Tau normalized to total Tau using ImageJ.

**Supplementary** **Fig. 3.** Inhibition of Mre11 prevents DRGN apoptosis and stimulates neurite outgrowth in the presence of inhibitory CME. (**A**) Adult DRGN stained at 4 days after plating, with anti-γH2Ax antibodies to demonstrate double strand breaks (arrows) in DRGN *in vitro*, whilst satellite glial cells did not possess double strand breaks (arrowheads). (**B**) Representative images from DRGN after 4 days in culture and treated with vehicle, FGF2 (positive control), mirin and KU-60019. (**C-E**) Mirin or KU-60019 treatment enhances (**C**), DRGN survival, (**D**) % DRGN with neurites and (**E**) increased the mean length of the longest neurite. Comparison in (C-E) by one-way ANOVA with Dunnett’s post-hoc test, n = 3 wells/condition, 3 independent repeats (total n = 9 wells/condition (all 9 data points shown on graphs)). Scale bars in **A** = 100µm and **B** = 50µm.

**Supplementary Fig. 4.** Intra-DRG injection of DNA plasmids to knock down Mre11 and ATM using non-viral *in vivo*-JetPEI (PEI) promotes significant functional repair after DC injury *in vivo*. (**A**) PEI delivered plasmids significantly knock down Mre11 and ATM mRNA expression in spinal L4/L5 DRGs at 4 weeks after DC injury. (**B**) Immunohistochemistry for γH2Ax to demonstrate DNA breaks 4 weeks after DC injury and their attenuation by shMre11 and shATM in DRGN. (**C**) Quantification of γH2Ax+ pixels/cells to demonstrate significant attenuation of double strand breaks by shMre11 and shATM. (**D**) Negative CAP amplitudes were significantly attenuated in DC+vehicle-treated rats but were significantly improved in DC+PEI-shMre11 and DC+PEI-shATM treatment (P<0.0001, one-way ANOVA (main effect)). (**E**) Mean CAP area at different stimulation intensities were significantly attenuated in DC+vehicle-treated rats but improved significantly in DC+PEI-shMre11 and DC+PEI-shATM-treated rats (P<0.0001, one-way ANOVA (main effect)). (**F**) Mean tape sensing and removal times are restored to normal 3 weeks after treatment with PEI-shMre11 and PEI-shATM (P<0.0001, independent sample t-test (DC+vehicle vs DC+PEI-shMre11/DC+PEI-shATM at 3 weeks) whilst a significant deficit remains in DC+vehicle-treated rats (# = P<0.00011, generalized linear mixed models over the whole 6 weeks). (**G**) Mean error ratio to show the number of slips vs. total number of steps in the horizontal ladder walking test also returns to normal 3 weeks after treatment with PEI-shMre11 and PEI-shATM (P<0.0001, independent sample t-test (DC+vehicle vs DC+PEI-shMre11/DC+PEI-shATM at 3 weeks)), with a deficit remaining in DC+vehicle-treated rats (## = P<0.00014, linear mixed models over the whole 6 weeks). AU = arbitrary units. n = 6 rats/treatment/test, 3 independent repeats (total n = 18 rats/treatment/test). Scale bars in **B** = 50µm, insets in **B** = 10µm.

**Supplementary Table 1.** Extended Statistics to show degrees of freedom (df), F and actual P values.

| **Figure** | **Exp. unit** | **Degrees of freedom** | **F** | **Actual P value** |
| --- | --- | --- | --- | --- |
| Fig. 1B | Fly | 15 | 51.3 | 0.0001 |
| Fig. 1C | Fly | 3, 586 | 36.8 | 0.0001 |
| Fig. 1E | Fly | 4, 122 | 15.9 | 0.0001 |
| Fig. 2A | Fly | 3 | 29.1 | 0.0001 |
| Fig. 2B (PDF+) | Fly | 2 | 46.0 | 0.0001 |
| Fig. 2B (PDF-) | Fly | 2 | 42.1 | 0.0001 |
| Fig. 2D | Fly | 3 | 14.5 | 0.0023 |
| Fig. 3B | Blots | 3 | 2596.7 | 0.0001 |
| Fig. 3C | Wells | 3 | 467.3 | 0.0001 |
| Fig. 3E | Wells | 3 | 2984.3 | 0.0001 |
| Fig. 3F | Wells | 3 | 1029.5 | 0.0001 |
| Fig. 4B | Blots | 2 | 1790.8 | 0.0001 |
| Fig. 4D | Blots | 2 | 421.0 | 0.0001 |
| Fig. 4F | Retinae | 3 | 2118.9 | 0.0001 |
| Fig. 4H | Nerves | 2 | 5430.0 | 0.0001 |
| Fig. 5C | Rats | 2 | 702.3 | 0.0001 |
| Fig. 5E | Blots | 2 | 198.4 | 0.0001 |
| Fig. 6B | Rats | 2 | 780.0 | 0.0001 |
| Fig. 7C | Rats | 3 | 3023.1 | 0.0001 |
| Supplementary Fig. 2B | Fly | 3, 586 | 2.842 | 0.0372 |
| Supplementary Fig. 2E | Fly | 3 | 28.70 | 0.0001 |
| Supplementary Fig. 3C | Wells | 3 | 623.7 | 0.0001 |
| Supplementary Fig. 3D | Wells | 3 | 1063.8 | 0.0001 |
| Supplementary Fig. 3E | Wells | 3 | 1159.9 | 0.0001 |
| Supplementary Fig. 4A | Rats | 2 | 812.7 | 0.0001 |
| Supplementary Fig. 4C | Rats | 2 | 407.9 | 0.0001 |
| Supplementary Fig. 4E | Rats | 3 | 648.1 | 0.0001 |

Full (non-cropped) western blots (including molecular weight markers) are shown below, corresponding to the cropped blots shown in the specified figures in the main article. Cropped regions in the main article are indicated by the red box.
